# Chromatic‐Invariant Photothermal Fabrics Enabled by Narrow‐Bandgap Organic Semiconductors for Wearable Solar Energy Harvesting

**DOI:** 10.1002/adma.202519334

**Published:** 2026-03-22

**Authors:** Jingshuai Zhu, Jianming Chen, Shiyu Liao, Jiaxin Zheng, Shiguo Chen, Yuanfeng Wang, Xungai Wang

**Affiliations:** ^1^ College of Materials Science and Engineering Shenzhen University Shenzhen China; ^2^ Joint Research Centre for Fiber Innovations and Renewable Materials (JRC‐FIRM) School of Fashion and Textiles The Hong Kong Polytechnic University Kowloon Hong Kong China; ^3^ Research Institute for Intelligent Wearable Systems (RI‐IWEAR) School of Fashion and Textiles The Hong Kong Polytechnic University Kowloon Hong Kong China; ^4^ School of Advanced Materials Peking University Shenzhen Graduate School Shenzhen China; ^5^ Zhejiang Sci‐Tech University College of Textile Science and Engineering Hangzhou China

**Keywords:** chromatic‐invariant, fabric thermal management, near‐infrared absorption, organic semiconductor, photothermal molecules

## Abstract

The increasing popularity of outdoor activewear has placed higher demands on its thermal comfort, multi‐functionality, and esthetics. Existing photothermal fabrics for cold weathers are based on visible light absorption, which limits color design options. Herein, a chromatic‐invariant photothermal material is developed and integrated onto fabrics to prepare novel photothermal clothing, achieving 4°C–8°C temperature increase within only 5 min under 1000 W m^−^^2^ simulated solar irradiation. It exhibits strong absorption in the near‐infrared region and effectively converts the radiation into thermal energy, while maintaining the original visual colors of fabrics due to high reflectivity across the visible spectrum. Additionally, this photothermal material does not compromise the original functionality of fabrics and is suitable for application on various types of fabric materials. Furthermore, the prepared fabric achieves a sterilization rate exceeding 95.3% after only 20 min of irradiation under 1000 W m^−^^2^ standard simulated solar light to ensure hygiene during outdoor activities. These results demonstrate a versatile wearable strategy with integrated functionality and aesthetic appeal for maintaining personal thermal comfort in cold‐weather outdoor scenarios.

## Graphical abstract

A chromatic‐invariant photothermal fabric is fabricated for outdoor activewear, realizing 4°C–8°C heating within 5 min under 1000 W m^−^^2^ illumination via NIR absorption, retaining high visible‐light reflectivity and original fabric functions, and achieving >95.3% sterilization in 20 min.

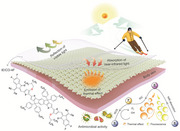

## 1 Introduction

Maintaining body temperature and physiological thermal equilibrium in extremely cold outdoor environments is crucial for survival and activities, including sports and recreation, healthcare, and specialized occupations [1, 2, 3, 4, 5]. Given the near‐universal availability and high energy density of solar irradiation in outdoor settings, the development of efficient solar‐to‐thermal energy conversion textiles to harness the ambient solar energy presents a critical strategy for addressing this challenge [6, 7, 8, 9, 10, 11, 12]. The currently used photothermal materials in textiles, including carbon‐based materials, metallic nanoparticles, metal oxides, and so on [13, 14, 15, 16, 17, 18], demonstrate broadband solar absorption, resulting in a characteristic dark appearance [19, 20, 21, 22, 23]. This presents a significant challenge for achieving vibrant colors in the activewear, thereby limiting its aesthetic appeal.

To address this challenge, researchers have discovered that the transparent hollow hairs of polar bear fur, which reflect sunlight, create a photothermal material with a white appearance [24, 25, 26, 27]. However, replicating such hollow structures artificially has always been a challenge, and they often appear in monotonous colors [28, 29]. Researchers embedded cesium‐tungsten bronze nanoparticles within nylon fibers to create a white fabric with excellent photothermal heating properties. However, the limitation of this fabric lies in the specific physical structure of its material, which must be fabricated into nanoparticles, thereby restricting its application range from a process perspective [30]. Notably, near‐infrared (NIR) radiation constitutes over 50% of the total solar energy, representing a key source for thermal energy harvesting [31, 32, 33, 34]. In this regard, organic semiconductor‐based photothermal materials offer a promising solution. Through molecular engineering, their absorption profiles can be precisely tailored to exhibit intense NIR absorption while maintaining minimal visible‐light absorption [35, 36, 37, 38, 39, 40, 41, 42, 43]. This selective absorption characteristic endows them with substantial potential for application in chromatic‐invariant thermal management textiles.

**FIGURE 1:**
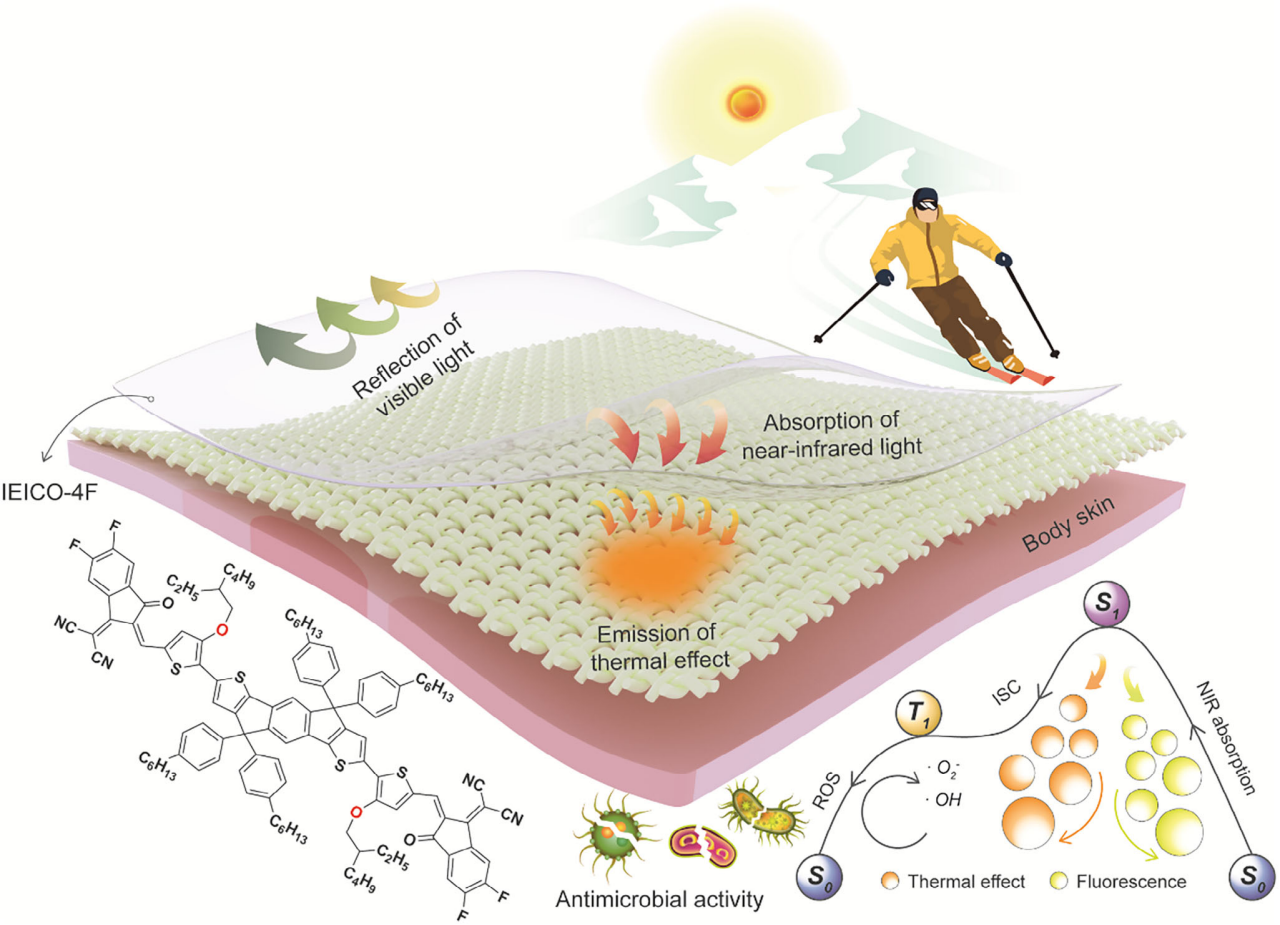
Schematic of chromatic‐invariant photothermal fabrics enabled by narrow‐bandgap organic semiconductors for wearable solar energy harvesting.

This study presents a simple solution dip‐coating method to prepare photothermal fabrics using the synthesized narrow‐bandgap organic semiconductor IEICO‐4F, enabling chromatic‐invariant wearable solar energy harvesting (Figure 1). The oxygen‐containing bridge end‐group in IEICO‐4F induces a narrower bandgap and a red‐shift compared to conventionally used oxygen‐free IEIC‐4F, resulting in enhanced photothermal performance. More importantly IEICO‐4F with its narrow bandgap, exhibits very weak absorption in the visible region, while its absorption peak (852 nm) lies in the near‐infrared region. This selective absorption ensures high reflection in the visible range (with up to 84% reflectivity) and effective light‐to‐heat conversion in the near‐infrared region. Moreover, the low‐loading IEICO‐4F coating (0.25 g m^−2^), formed via a simple dip‐coating process, is sufficiently thin to avoid significant visible light absorption, thereby preventing noticeable changes in the fabric's appearance. The prepared cotton‐IEICO‐4F composite fabrics (with both dark and light colors) demonstrate a 4°C–8°C temperature increase over blank cotton fabrics under 1000 W m^−2^ simulated sunlight within only 5 min. Furthermore, the organic semiconductor‐integrated fabrics maintain key textile properties, including fabric morphology, air permeability, and moisture permeability, compared to blank cotton fabrics. Moreover, this photothermal material works on diverse fabrics including cotton, silk, polyester, and nylon, achieving heat generation with excellent color stability. Concurrently, these fabrics display exceptional antibacterial efficacy under simulated sunlight within 20 min, achieving 95.3% and 81.7% inhibition rates against *Staphylococcus aureus* and *Escherichia coli*, respectively. Compared with blank cotton fabrics, IEICO‐4F fabrics integrate higher solar‐to‐thermal conversion (4°C–8°C temperature increase within 5 min under 1000 W m^−^^2^), chromatic invariance (Δ*E*: 0.71–2.85), >95% antibacterial rate against *Staphylococcus aureus*, and uncompromised textile properties (air permeability, moisture permeability, and tensile strength), making them suitable for wearable thermal management in cold outdoor scenarios. Additionally, the analysis of chromatic‐invariant fabrics’ global application potential reveals a significant increase in photothermal temperature in both cold regions and sunny areas, further demonstrating the broad application scenarios.

## 2 Results and Discussion

**FIGURE 2:**
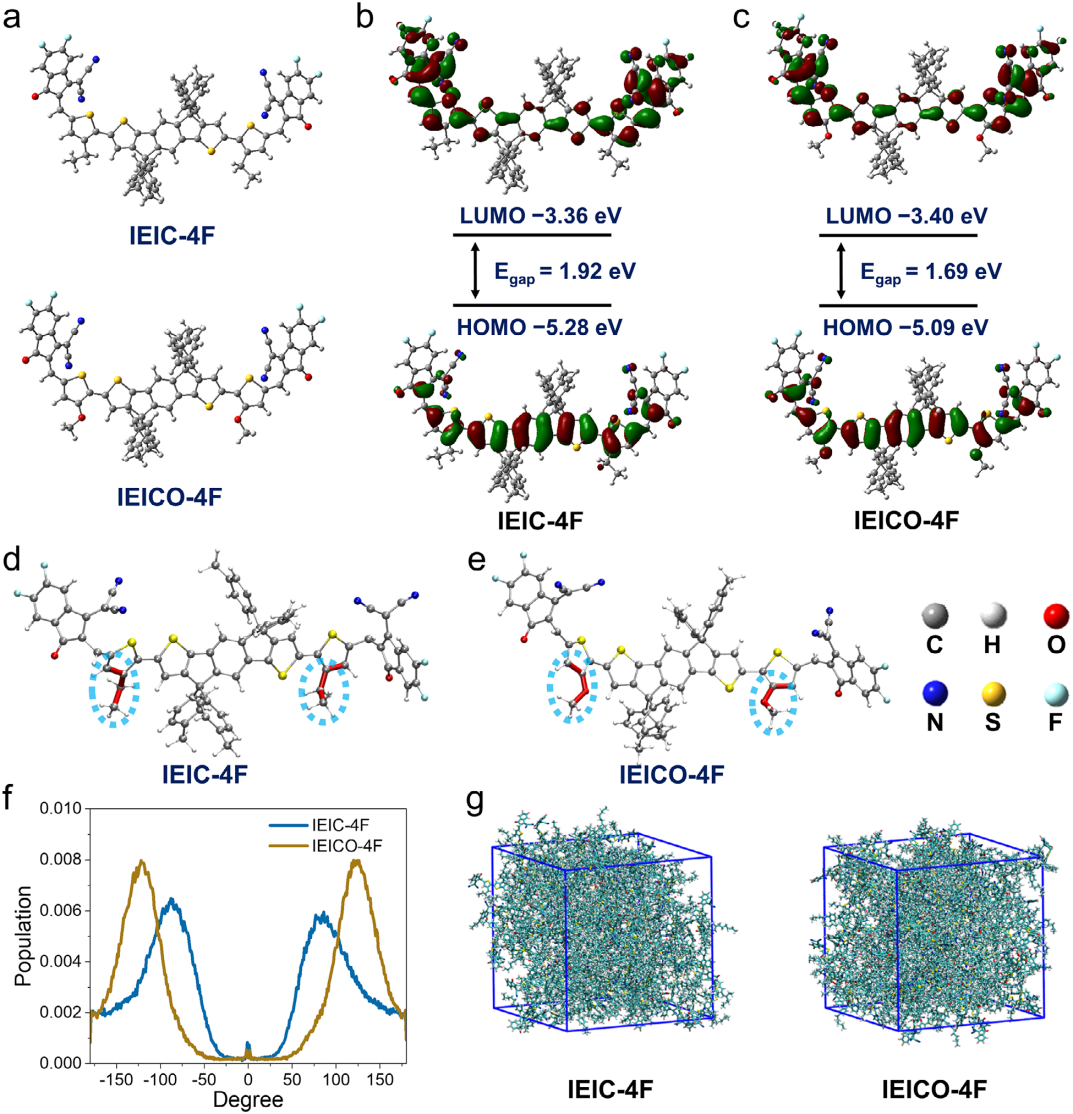
(a) Optimized ground‐state geometry for IEIC‐4F and IEICO‐4F. (b) Calculated LUMO and HOMO for IEIC‐4F and (c) IEICO‐4F. (d) Schematic illustration of IEIC‐4F, (e) and IEICO‐4F 3D structure. (f) Dihedral angle distributions of IEIC‐4F and IEICO‐4F in the aggregated state derived from MD simulations. (g) MD simulation of the structure of IEIC‐4F and IEICO‐4F.

**SCHEME 1:**
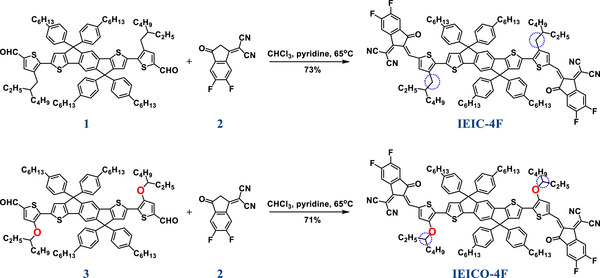
Synthetic route of IEIC‐4F and IEICO‐4F.

The photothermal molecules IEIC‐4F and IEICO‐4F are both conjugated compounds featuring a comparable planar structure (Figure 2a). They were synthesized via a Knoevenagel condensation reaction, and their detailed synthetic routes and preparation methods are depicted in Scheme 1. 1H NMR spectroscopy shows IEIC‐4F has a characteristic peak at *δ* 2.79 (m, 4H) for ─CH_2_− on the alkyl chain of bridged thiophene (absent in IEICO‐4F), while IEICO‐4F exhibits a unique peak at *δ* 4.11 (m, 2H) for ─O─CH─ (absent in IEIC‐4F), confirming its oxygen‐containing bridge. MS (MALDI‐TOF) corroborates this structural difference: measured m/z 1776.1 (IEIC‐4F) and 1779.6 (IEICO‐4F) match theoretical values, with the ∼4 mass difference due to IEICO‐4F's oxygen‐containing bridge vs. IEIC‐4F's oxygen‐free alkyl chain. Density functional theory (DFT) calculation was adopted to determine the energy levels of IEIC‐4F and IEICO‐4F. Figure 2b,c display the optimized molecular structures and the density of states distributions for the lowest unoccupied molecular orbital (LUMO) and highest occupied molecular orbital (HOMO), respectively. Compared with IEIC‐4F, IEICO‐4F exhibits a lower LUMO energy level and a higher HOMO energy level, resulting in a narrower energy bandgap (*E*_gap_). Solution‐phase absorption spectra exhibit distinct absorption profiles with high molar extinction coefficients (1.2 × 10^5^ m ^−1^ cm^−1^ at 770 nm for IEIC‐4F; 1.2 × 10^5^ m ^−1^ cm^−1^ at 863 nm for IEICO‐4F, Figure S1). The 93 nm red shift in IEICO‐4F absorption broadens its solar spectral response range. The optical bandgaps of IEIC‐4F and IEICO‐4F are respectively 1.41 and 1.28 eV, calculated from their corresponding absorption edges (Figure S2). The bandgap of IEICO‐4F is narrower than that of IEIC‐4F, which is attributed to the induced effect of oxygen atoms in its molecular structure. Consistent with the energy‐gap law, a smaller energy gap promotes non‐radiative decay, thus boosting heat production (Figure 1). Thus, IEICO‐4F potentially has more advantageous photothermal properties. The photoluminescence quantum yield (PLQY) results of IEIC‐4F and IEICO‐4F show that the PLQY of the IEIC‐4F thin film is 1.01%, while the PLQY of the IEICO‐4F thin film is as low as 0.31%. The decrease in the PLQY of IEICO‐4F confirms the enhanced non‐radiative decay effect induced by the narrow bandgap of IEICO‐4F. As illustrated in Figures S3 and S4, ultraviolet photo‐electron spectroscopy (UPS) measurements verify HOMO levels at −5.50 eV (IEIC‐4F) and −5.30 eV (IEICO‐4F); when combined with the optical bandgap, the calculated LUMO values are −4.09 and −4.02 eV, respectively, which align with DFT trends, indicating that the bandgap of IEICO‐4F (1.28 eV) was narrower than that of IEIC‐4F (1.41 eV). Thermal data from thermogravimetric analysis (TGA) of IEIC‐4F and IEICO‐4F are shown in Figures S5 and S6, respectively. Both materials can withstand temperatures exceeding 300°C, demonstrating their outstanding thermal stability and confirming their suitability for use as photothermal materials.

Subsequently, molecular dynamics (MD) simulations were further performed on individual IEIC‐4F and IEICO‐4F molecules as well as their aggregates. The MD simulation results indicate that, compared to IEIC‐4F, IEICO‐4F exhibits a stronger rotational behavior in its dihedral angle. The improved rotational freedom of the oxygen‐containing dihedral angle provides greater conformational flexibility, which promotes non‐radiative decay. This is evidenced by the lower PLQY of IEICO‐4F compared to IEIC‐4F. This property facilitates rapid conformational changes under photothermal stimulation, suggesting superior thermal properties for IEICO‐4F [44]. This is because IEIC‐4F employs an alkyl chain (─C─C─) on the bridged thiophene, while IEICO‐4F uses an alkoxy chain (−O─C─) as a replacement on its bridged thiophene. The van der Waals radius of the oxygen atom is smaller than that of the carbon atom, and the C─O bond length in IEICO‐4F is shorter than the C─C bond length in IEIC‐4F. This structural feature reduces the spatial crowding between the terminal indanone units and the central conjugated backbone, lowering the “hindrance” during dihedral angle rotation. This conclusion is supported by the distribution of the key dihedral angle involving the oxygen atom (Figure 2d,e), which was tracked over the simulation time (Figure 2f). The two peaks in the distribution correspond to angles on either side of the molecule. Since this position is linked to atoms on the thiophene ring, which is planar and thus complementary to the dihedral angle, it remains relatively fixed. The magnitude of the angle increases with the absolute deviation from 0°. For a more intuitive understanding, a snapshot of the packing mode from the production simulation was selected to show the accumulation pattern of IEIC‐4F and IEICO‐4F molecules in the solid state. As shown in Figure 2g, IEIC‐4F and IEICO‐4F aggregates exhibit amorphous characteristics. This random stacking feature of molecules provides sufficient space for molecular movement, which leads to strong photothermal conversion.

**FIGURE 3:**
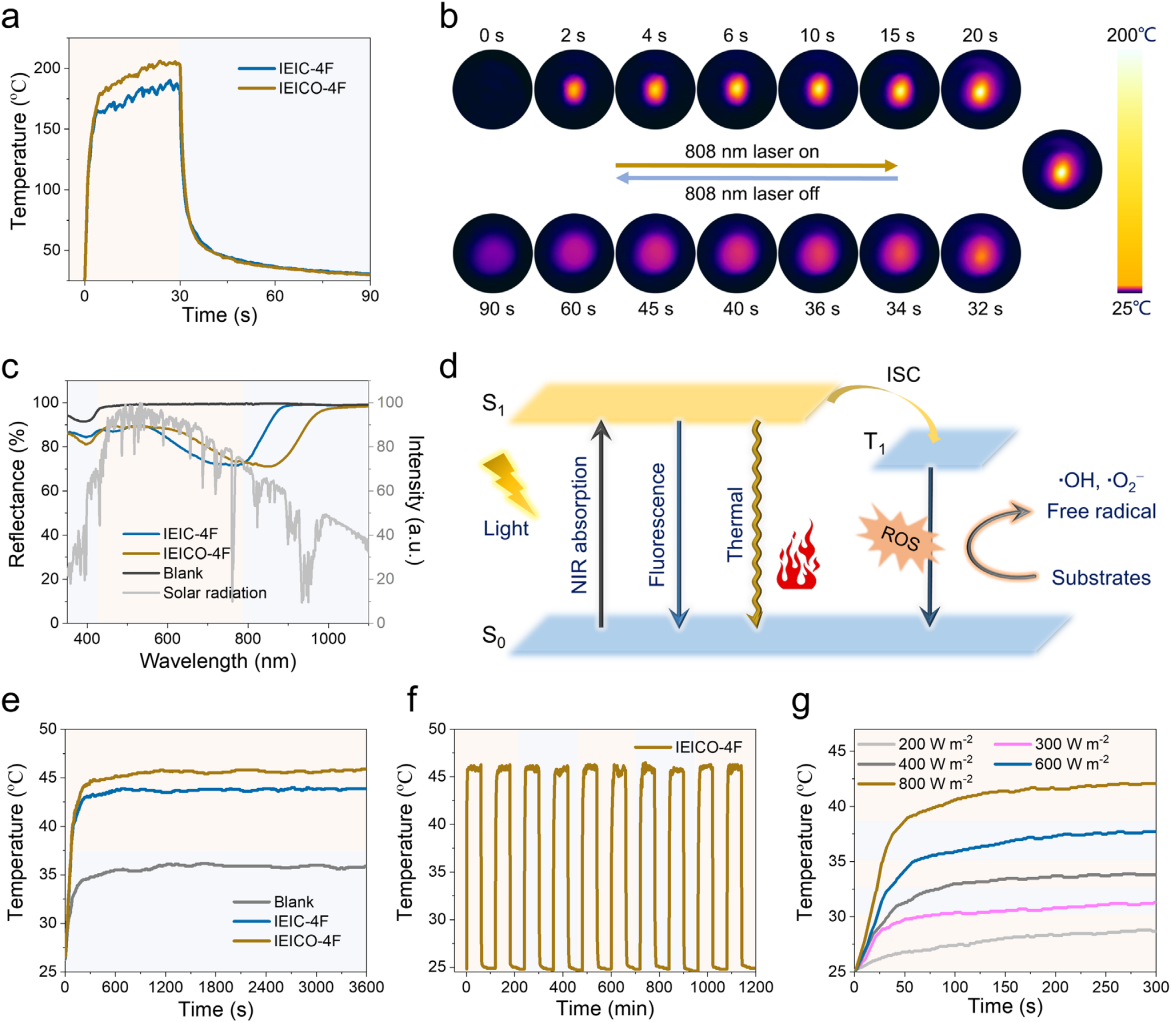
(a) Photothermal conversion behavior of IEIC‐4F and IEICO‐4F powder under 808 nm laser irradiation (10000 W m^−2^) measured by a thermocouple. (b) Infrared thermal photos of IEICO‐4F powder taken by a thermal imager under 808 nm laser irradiation (10000 W m^−2^), with the laser turned off at 30 s. (c) Light reflection spectra of IEIC‐4F and IEICO‐4F fabrics and solar spectral intensity (AM 1.5 G). (d) Working diagram of photothermal materials. (e) Photothermal temperature of blank, IEIC‐4F, and IEICO‐4F fabrics (4 × 4 cm) under one sun irradiation measured by a thermocouple. (f) Photothermal cycling curve of IEICO‐4F fabric under one sun irradiation measured by a thermocouple. (g) Curves of irradiation time and fabric temperature under different light intensities measured by a thermocouple.

Rapid heating is an essential property of photothermal materials. To investigate the intrinsic photothermal properties of organic photothermal molecules, the solid‐state photothermal conversion temperatures of IEIC‐4F and IEICO‐4F under laser irradiation were measured. As shown in Figure 3a,b, under laser irradiation, both solid IEIC‑4F and IEICO‑4F can rapidly raise their temperature to above 180 °C within 30 s, indicating their excellent photothermal conversion ability. Moreover, through calculations (Figures S7–S11), the photothermal conversion efficiency (*η*) of IEICO‐4F is 41.3%, which is higher than that of IEIC‐4F at 40.7%. Upon turning off the laser at 30 s, the temperature gradually cooled back to room temperature. After thoroughly dissolving the organic photothermal materials, the solution was coated onto cotton fabrics by a simple dipping method, and the photothermal properties of the coated fabrics were investigated. As shown in Figure 3c, compared with the IEIC‐4F fabric (0.25 g m^−2^), the reflection spectrum of the IEICO‐4F fabric (0.25 g m^−2^) shows a significant red shift, which facilitates enhanced absorption of near‐infrared light and subsequent heat generation. These organic semiconductor fabrics, which have high reflectivity in the visible light region and can absorb light in the near‐infrared region to generate heat, provide a possible solution to imparting photothermal properties to textiles without altering their color. Additionally, based on absorbance measurements (Figures S1 and S2 and S12), the calculated solar‐weighted absorptance (SWA) values for IEICO‐4F in solution, as a film, and on cotton fabric within the 350–1100 nm spectral range are 0.130, 0.094, and 0.079, respectively. These values are higher than those of IEIC‐4F in solution (0.128), as a film (0.076), and on cotton fabric (0.074). This indicates that under the solar spectrum conditions, the light absorption capacity of IEICO‐4F is stronger than that of IEIC‐4F.

The photothermal action mechanism of IEIC‐4F and IEICO‐4F fabrics is shown in Figure 3d. Based on energy‐gap law, the photothermal conversion behavior of IEIC‐4F and IEICO‐4F fabrics was further investigated. The photothermal conversion curves show that under the standard simulated solar radiation condition of 1000 W m^−2^, the IEIC‐4F and IEICO‐4F fabrics quickly reached the optimal equilibrium temperatures under the effect of rapid heating (Figure 3e). Under the same conditions, measurements taken with a contact thermocouple showed that the IEIC‐4F fabric reached a temperature of 43.0°C within 5 min, while the IEICO‐4F fabric attained 44.7°C, exceeding that of IEIC‐4F. In contrast, the temperature of the blank cotton fabric was only 35.2°C. After half an hour of illumination, the temperature reached its saturation point. Furthermore, over a one‐hour period, the photothermal temperature of the IEICO‐4F fabric could be consistently maintained above 45°C (Figure 3e). Compared to the blank cotton fabric, the IEICO‐4F fabric requires only 0.25 g m^−2^ of photothermal material to achieve a temperature increase of approximately 26%, demonstrating excellent material economy (The temperature of cotton fabric with varying IEICO‐4F mass per unit area is shown in Figure S13. To standardize the experimental variables, 0.25 g m^−2^ was selected). Moreover, a cyclic experiment was conducted on the IEICO‐4F fabric, involving repeated photothermal heating and cooling under simulated solar radiation at an intensity of 1000 W m^−2^ (Figure 3f). Using a contact thermocouple, the temperature was recorded over a 20‐h period. In every cycle, the temperature profile remained similar, signifying the outstanding stability of the IEICO‐4F fabric. A comparative color difference analysis was conducted between the IEICO‐4F fabric samples before (L*: 76.68, a*: 8.27, b*: −2.08) and after (L*: 76.51, a*: 8.42, b*: −1.97) the 20‐hour cyclic testing. The results showed a very small color change, with a Δ*E* value of 0.25, indicating excellent color stability. The temperature change of the IEICO‐4F fabric under varying light intensities was examined, as presented in Figure 3g and Table S1. As light intensity rose (from 200 to 2000 W m^−2^, within 5 min), the temperature of the IEICO‐4F fabric increased steadily. It was observed that light intensity has an almost linear relationship with saturation temperature (Figures S14 and S15), so in practical wearable scenarios, the fabric temperature is controllable, and the loading mass can be adjusted on demand to adapt to different environments.

**FIGURE 4:**
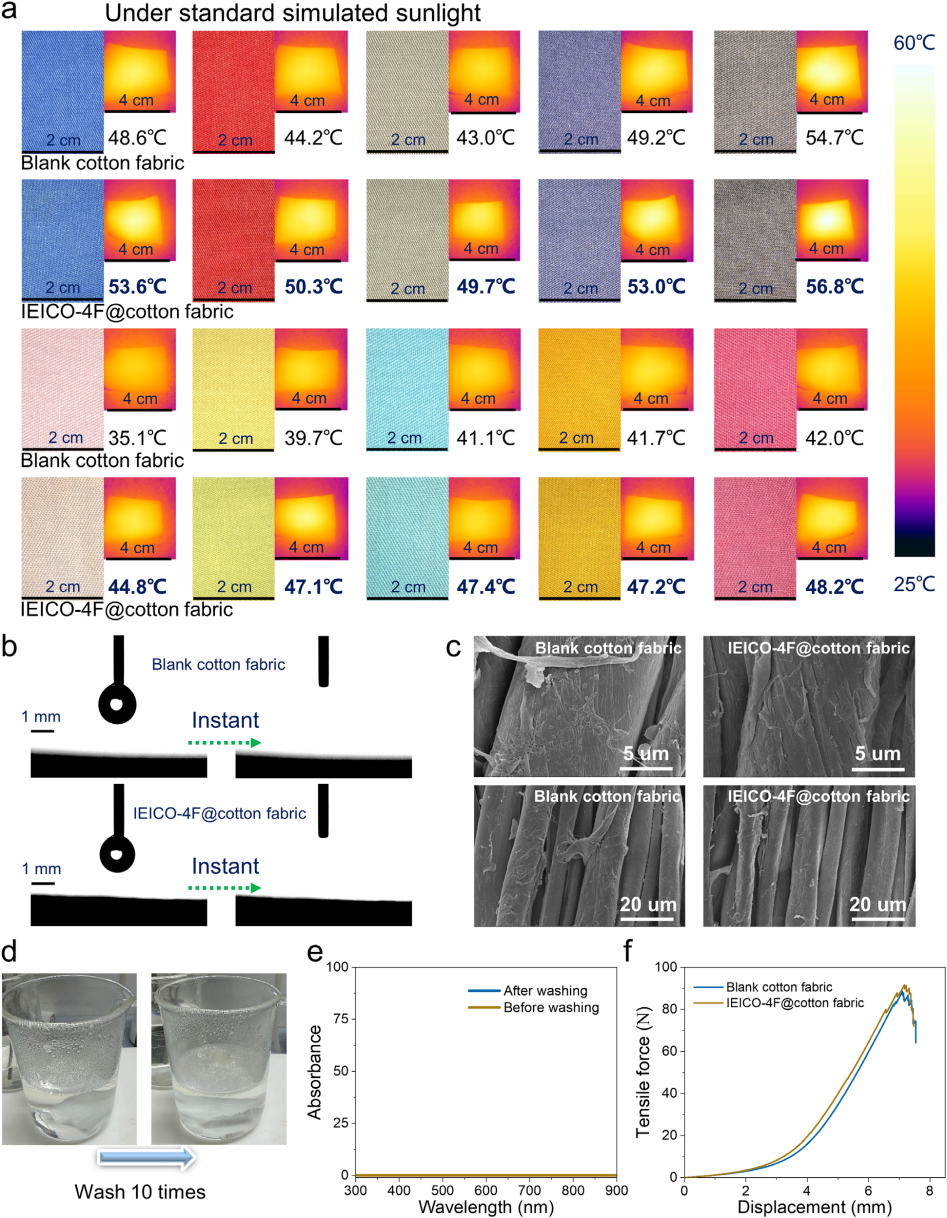
(a) Different colored fabrics under one standard sunlight irradiation (the temperature is the average value of the three experiments), the images were captured by a thermal imager, and the temperature values were recorded by a thermocouple. (b) Contact angle images of water on cotton fabric. (c) SEM images of blank cotton fabric and IEICO‐4F@cotton fabric at magnifications of 20000X and 5000X. (d) Photo of IEICO‐4F@cotton fabric stirred in detergent (at 65°C for 10 times, 30 min each time). (e) Absorption spectrum of detergent before and after stirring. (f) Tensile curve of the blank cotton fabric and IEICO‐4F@cotton fabric.

To visually investigate the color maintaining of IEICO‐4F, it was combined with different colored cotton fabrics, and the color changes of these fabrics were observed under 1000 W m^−2^ simulated solar radiation conditions. As shown in Figure 4a, both dark and light‐colored cotton fabrics loaded with the IEICO‐4F show negligible color change compared to the blank fabrics. In Table S2, the total color difference (Δ*E*) values between IEICO‐4F@cotton and blank cotton of various colors are all below 3, with the maximum value of only 2.85 for white samples. The Δ*E* values for other colors range from 0.71 to 2.43. These results clearly indicate that the loading of IEICO‐4F has no significant impact on the color of the original cotton fabrics. More importantly, the fabrics loaded with the IEICO‐4F photothermal material reached a temperature of 4°C–8°C higher than that of blank cotton fabrics within only 5 min under 1000 W m^−2^ standard simulated solar radiation. Then, the photothermal properties of IEICO‐4F when loaded onto different types of fabrics were further studied (Figure S16). In addition to cotton, other fabrics (such as silk, polyester and nylon) loaded with IEICO‐4F showed no significant color change and it can increase the temperatures by 6°C–8°C under standard simulated sunlight exposure within 5 min. These results demonstrate the feasibility of the IEICO‐4F organic semiconductor for chromatic‐invariant wearable thermal management textile applications.

Furthermore, the conventional physical properties of the IEICO‐4F‐loaded fabrics were also investigated. As shown in Figure 4b, the IEICO‐4F treated cotton fabric (IEICO‐4F@cotton fabric) exhibits superhydrophilicity, with a water contact angle consistent with that of blank cotton fabric (0°). This indicates that the observed hydrophilicity originates from the cotton substrate itself, and the thin IEICO‐4F functional layer does not alter its inherent wetting property. According to the microscopic images provided by SEM (Figure 4c), it can be observed that compared to the blank cotton fabric, the IEICO‐4F@cotton fabric also exhibits a well‐preserved fabric morphology. The static secondary ion mass spectrometry (TOF‐SIMS) measurement was employed to investigate the distribution of IEICO‐4F material on the cotton fabrics, and the ions F^−^, CN^−^, and S^−^ were used as indicators for IEICO‐4F. Figure S17 shows that the distribution of F^−^, CN^−^, and S^−^ in the cotton fabric coated with IEICO‐4F is uniform. As shown in Table 1, the air permeability of the blank cotton fabric is 18.27 cm^3^/s/cm^2^, compared to 18.02 cm^3^/s/cm^2^ for the IEICO‐4F@cotton fabric. Similarly, its moisture permeability measures 4910.25 g/m^2^·day versus 4816.96 g/m^2^·day for the treated fabric. Therefore, no significant change was observed for the blank and modified cotton fabrics in terms of air and moisture permeability. These results indicate that the cotton fabrics loaded with organic photothermal materials still retain their original physical properties. The color fastness of IEICO‐4F@cotton fabric was also studied. As shown in Table S3, the color fastness against water, laundry detergent, perspiration, and rubbing of IEICO‐4F@cotton fabric all met the respective standards (GB/T 5713‐2013, GB/T 3921‐2008, GB/T 3922‐2013, GB/T 3920‐2008). Additionally, after stirring the IEICO‐4F@cotton fabric in detergent‐containing water at 65°C for 10 cycles, with each cycle lasting 30 min, the spectrum of the washed water showed no change (Figure 4d,e). This indicates that no organic semiconductor material was leaching during the washing process. After the IEICO‐4F@cotton fabric, IEICO‐4F@silk, IEICO‐4F@polyester, and IEICO‐4F@nylon were removed and dried after ten washing cycles, the photothermal temperature, averaged over three measurements, remained above 44°C within 5 min, further demonstrating the excellent adhesion strength and thermal stability of the IEICO‐4F@cotton fabric (Table S4). Further studies were conducted on the stability of IEICO‐4F fabrics after an increased number of washing and drying cycles. It was found that after 30 washing and drying cycles, the photothermal temperature decreased, which may be attributed to the absence of a binder (Table S4). The reason for the relatively strong binding between the IEICO‐4F and cotton fabric maybe as follows: The cyano and carbonyl groups in the IEICO‐4F molecule form hydrogen bond interactions with the hydroxyl groups on the cellulose surface; IEICO‐4F penetrates into the micro‐nano pore structures on the cotton fiber surface, forming mechanical anchoring after drying to prevent washing‐induced detachment; the planar structure of the IEICO‐4F molecule enables *π–π* stacking, resulting in a continuous and dense film that tightly adheres to the fiber surface; at low loading levels, the coating is thin and uniform, reducing internal stress and enhancing adhesion stability. As can be seen from the tensile curve in Figure 4f, the tensile strength of the IEICO‐4F@cotton fabric does not show a significant change compared to the blank cotton fabric. These further demonstrate the advantage of integrating organic semiconductors onto cotton fabrics without affecting their original properties. Obviously, the IEICO‐4F@cotton fabric can keep the body warm without the need of thicker, heavier and darker clothing or heating regulators, thus having broad application potential in cold environments.

**TABLE 1:** The air permeability and moisture permeability of fabrics.

	Air permeability	Moisture permeability
Blank cotton fabric	18.27 cm^3^/s/cm^2^	4910.25 g/m^2^·day
IEICO‐4F@cotton fabric	18.02 cm^3^/s/cm^2^	4816.96 g/m^2^·day

**FIGURE 5:**
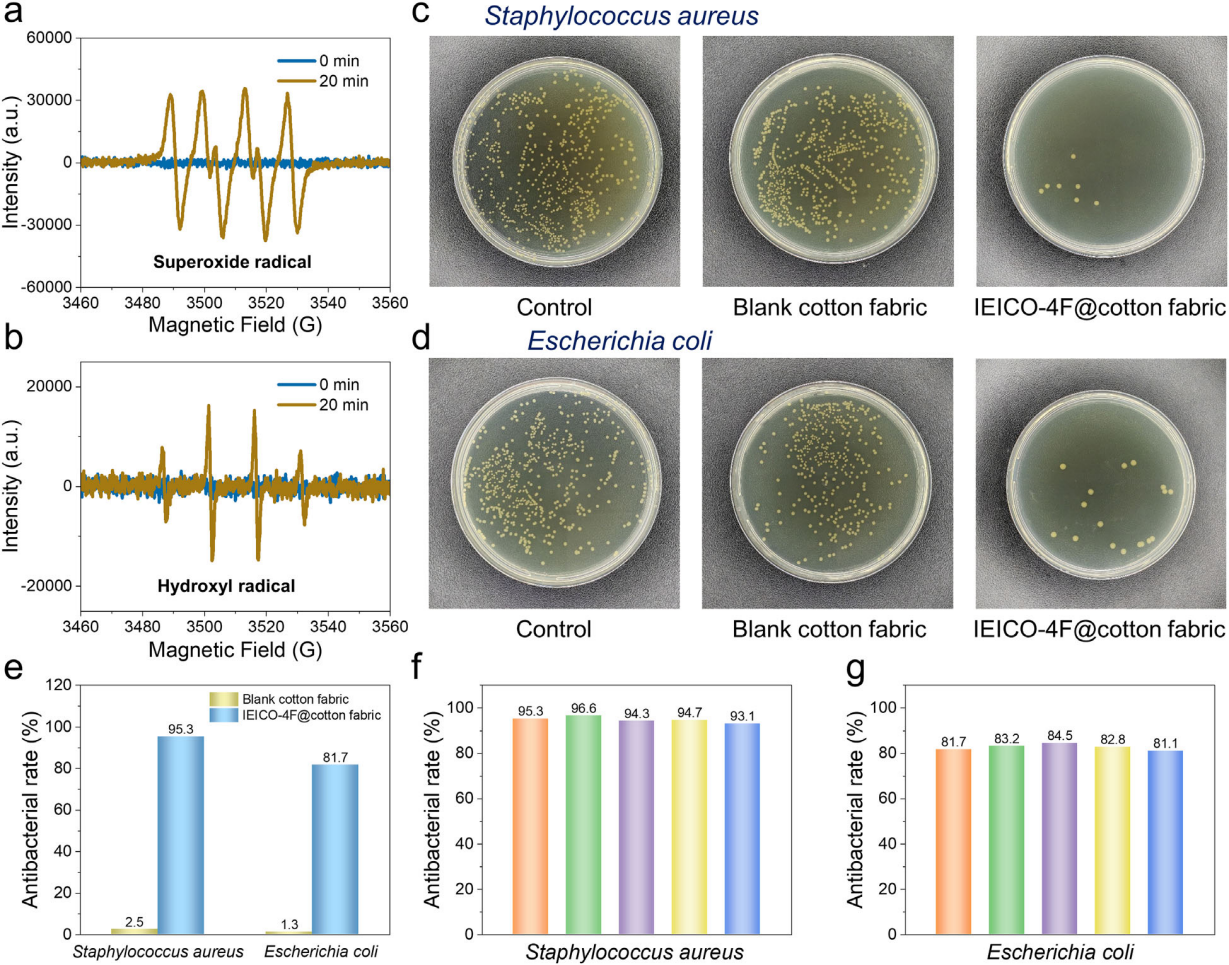
(a) ESR spectra of superoxide radical and, (b) hydroxyl radical of IEICO‐4F@cotton fabric in the dark and after 20 min irradiation. (c) Representative images of colony forming units for *Staphylococcus aureus* and (d) *Escherichia coli* suspensions by spreading their media on LB agar plates incubation at 37°C. (e) The antibacterial rates of *Staphylococcus aureus* and *Escherichia coli*. (f) Cyclic antibacterial performance against *Staphylococcus aureus* and (g) *Escherichia coli* on the same IEICO‐4F@Cotton fabric.

Outdoor activities can foster bacterial growth, potentially leading to health issues and unpleasant odors. In this regard, organic semiconductor materials capable of generating Reactive Oxygen Species (ROS) upon light initiation hold promise for simultaneously addressing antimicrobial challenges in outdoor wear. Electron spin resonance (ESR) measurements were used to evaluate the ROS (superoxide radical and hydroxyl radical) production capacity of the IEICO‐4F@cotton fabric (Figure 3d). Both radicals generated strong signals upon light exposure, which means that the IEICO‐4F@cotton fabric has excellent photocatalytic antibacterial activity (Figure 5a,b). Here, both Gram‐positive *Staphylococcus aureus* and Gram‐negative *Escherichia coli* were selected to assess the antimicrobial properties of the fabrics with simulated solar irradiation. Cotton fabric functionalized with IEICO‐4F exhibited high bacterial inhibition rates of 95.3% against *Staphylococcus aureus* and 81.7% against *Escherichia coli*, compared to only 2.5% and 1.3%, respectively, for the blank fabric (Figure 5c–e; Table S5). Moreover, the antibacterial rates against both *Staphylococcus aureus* and *Escherichia coli* for the same IEICO‐4F@cotton fabric remained stable over five cycles under standard simulated sunlight (Figure 5f,g). We further investigated the antibacterial mechanism through antibacterial experiments. As shown in Table S6, the ROS scavenging experiments demonstrate that the scavenger exhibits no inherent antibacterial effect in the absence of light (eliminating interference); even when ROS are scavenged under light irradiation, the fabric still maintains strong antibacterial activity (the antibacterial rate against *Escherichia coli* was 77.7% and that against Staphylococcus aureus was 92.7%), confirming that ROS only play a synergistic role. Therefore, the antibacterial mechanism is as follows: IEICO‐4F achieves efficient antibacterial activity through the combined action of photothermal induced temperature increase and ROS generation under light irradiation. Subsequently, the antibacterial performance of the IEICO‐4F@cotton fabric under low‐light environments was evaluated. As presented in Table S6, even at a low light intensity of 500 W m^−^^2^, the fabric retains impressive antibacterial efficiencies of 79.8% against *Escherichia coli* and 94.9% against *Staphylococcus aureus*, respectively. Under weaker illumination of 300 W m^−^^2^, the antibacterial efficiencies against the two strains persist at 73.9% (*Escherichia coli*) and 90.8% (*Staphylococcus aureus*), respectively. These findings confirm that the IEICO‐4F@cotton fabric maintains superior antibacterial activity even under weak sunlight conditions encountered in practical wearable scenarios. In addition, the antibacterial activity of IEICO‐4F fabric after 20 h of light exposure was tested, and the fabric still maintained high antibacterial efficiencies of 79.2% against *Escherichia coli* and 92.5% against *Staphylococcus aureus*, respectively. These results demonstrate the exceptional antibacterial performance and wearable potential of organic semiconductor‐treated fabrics. We further evaluated the cytotoxicity of IEICO‐4F@cotton fabric using two common cell lines: human keratinocytes (HaCaT) and mouse fibroblasts (L929). As shown in Figure S18, the results showed that at lower extract concentrations (12.5%–50%), both cell types maintained favorable cell viability, with the viability being 75%–99% after 24, 48, and 72 h of incubation. However, at higher extract concentrations (100%), cell viability decreased to 60%–70%. Under light exposure, cell viability was 65%–80%, which may be attributed to the functional groups of the molecule itself and the generation of ROS.

**FIGURE 6:**
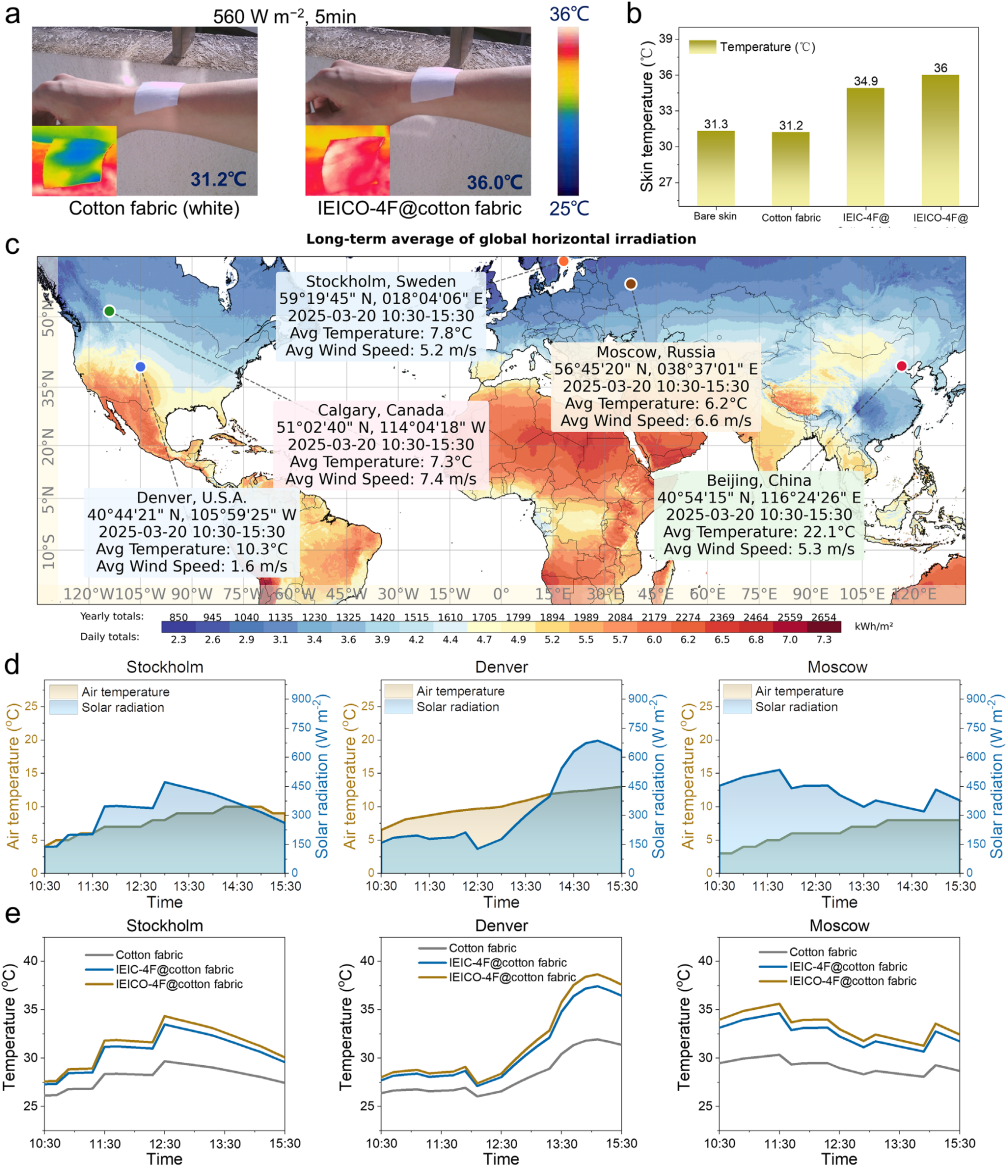
(a) Under the actual solar radiation of 560 W m^−2^, the thermal imaging diagram (Sunny, East wind level 2, Humidity 53%, Temperature 25°C, Hong Kong), the images were captured by a thermal imager, and the temperature values were recorded by a thermocouple, and (b) the temperature values of IEICO‐4F@cotton fabric on the human skin surface after 5 min of irradiation, recorded by a thermocouple. (c) Solar resource map of the world. Adapted under terms of the CC‐BY Creative Commons Attribution 4.0 International license(https://creativecommons.org/licenses/by/4.0) [45]. Marked points represent five test locations: Stockholm (Sweden), Denver (U.S.A.), Moscow (Russia), Beijing (China), Calgary (Canada), and the topographic information and meteorological information of these five test locations are provided in insets. (d) Detailed *I*_solar_ and *T*_ambient_ in Stockholm, Denver, and Moscow. (e) Real‐time temperature of the simulated skin covered by blank cotton fabric, IEIC‐4F@cotton fabric, and IEICO‐4F@cotton fabric over a duration of 5 h under sunlight in Stockholm, Denver, and Moscow.

Further research into the performance of photothermal fabrics under real‐world conditions is necessary. As shown in Figure 6a,b, the human skin was respectively covered with cotton fabric and IEICO‐4F@cotton fabric, upon exposure to real sunlight for 5 min. Their temperatures were monitored during this period. After 5 min of real solar exposure (∼560 W m^−2^), the surface temperature of bare skin and cotton fabric remained around 31°C, while that of IEIC‐4F@cotton fabric was 34.9°C, and the surface temperature of IEICO‐4F@cotton fabric reached 36°C, which was higher than that of the other fabrics. Under real low‐light conditions, the temperature of IEICO‐4F@cotton fabric was also higher than that of IEIC‐4F@cotton fabric, further verifying that introducing oxygen atoms in the photothermal molecular design can enhance photothermal performance. The observed exceptional solar heating function of IEICO‐4F@cotton fabric works worldwide under diverse weather conditions, as simulated in Stockholm (Sweden), Denver (U.S.A.), Moscow (Russia), Beijing (China), and Calgary (Canada) (Figure 6c). As shown in Figure 6d, in the cold environment of Stockholm (Sweden), Denver (U.S.A.), and Moscow (Russia) the average ambient temperature (*T*_ambient_) is approximately 7.8°C, 10.3°C, and 6.2°C, respectively, while the average solar radiation intensity (*I*_solar_) is approximately 322, 336, and 423 W m^−2^, respectively. The skin covered with IEICO‐4F@cotton fabric in the simulation reached an average temperature (*T*_ave_) of approximately 31.3°C, 31.6°C, and 33.3°C, respectively, which was about 4°C higher than that of the blank cotton fabric and 1°C higher than that of the IEIC‐4F@cotton fabric (Figure 6e). In inland areas, there is sufficient sunlight. For example, in Beijing (China) and Calgary (Canada), the *T*_ambient_ are approximately 22.1°C and 7.3°C, respectively, and the *I*_solar_ is approximately 712 and 539 W m^−2^, respectively (Figure S19). The IEICO‐4F@cotton fabric covered skin in the simulation reached a *T*_ave_ of approximately 39.1°C and 35.7°C, respectively, which was about 6°C higher than that of the blank cotton fabric and 1.5°C higher than that of the IEIC‐4F@cotton fabric (Figure S20). Obviously, IEICO‐4F@cotton fabric is capable of warming the human body without consuming non‐solar energy, whether in warm or cold environments. It is worth noting that the above results were achieved with an IEICO‐4F loading of 0.25 g m^−2^, and the degree of temperature increase can be tuned by varying the loading of IEICO‐4F in the fabric. The simulation data only considered the temperature rise of the photothermal fabrics under different sunlight intensities in various regions, without taking complex environmental factors into account. In future real‐world wear applications, it will be necessary to adjust the loading amount of the photothermal materials to better adapt to various scenarios.

## 3 Conclusion

In this work, a type of widely applicable chromatic‐invariant photothermal textile was proposed by incorporating the organic semiconductor IEICO‐4F within fabric substrates. When applied to both dark and light‐colored base fabrics, it can maintain the fabric color under sunlight because the reflection spectrum of the resulting textile closely matches the solar spectrum in visible light region (Δ*E*: 0.71–2.85). Only 0.25 g m^−2^ of IEICO‐4F on fabric can achieve a substantial temperature increase of 4°C–8°C within just 5 min under 1000 W m^−2^ standard solar irradiation. Furthermore, the treated fabrics exhibit excellent photo‐induced antibacterial properties against *Staphylococcus aureus* and *Escherichia coli*, while retaining desirable air and moisture permeability. These characteristics demonstrate the considerable potential of such organic semiconductor‐incorporated fabrics for wearable thermal management without sacrificing aesthetics. Future works may focus on tuning the molecular structures of organic semiconductors for enhanced near‐infrared absorption, engineering optimized fabric microstructures, and designing advanced functionalities—paving the way for more integrated smart and comfortable fabrics with outstanding performance.

## 4 Experimental Section

### 4.1 Chemicals and Materials

Dichloromethane and chloroform were purchased from Xilong Scientific Co. Ltd. Compound 1, Compound 2, and Compound 3 were purchased from Sunatech Inc. IEIC‐4F and IEICO‐4F were synthesized according to procedures reported in the literature [46, 47]. The LB medium was purchased from Qingdao Hi‐tech Industrial Park Hope Bio‐technology Co., Ltd.

### 4.2 Characterization and Molecular Simulations

All calculations were carried out with the Gaussian 09 software [48]. The geometries of molecules and solvation shells were optimized using the B3LYP level of theory in conjunction with the 6–311++G (d) basis set [49, 50]. Frequency analyses were done with the same basis set to confirm the obtained optimized stationary point.

Nuclear magnetic resonance (NMR) spectra were obtained with a Bruker 500 MHz spectrometer. Mass spectra measurements were carried out on a Bruker Daltonics Biflex III MALDI‐TOF Analyzer in the MALDI mode. Elemental analyses were conducted using a FLASH EA1112 elemental analyzer. Fourier transform infrared spectroscopy (FT‐IR) was performed using a Thermo Fisher Nicolet Is5 with a thin KBr pellet. Thermogravimetric analyses (TGA) was performed on a Netzsch STA449 F5 Jupiter instrument with a heating rate of 10 K min^−1^ under a nitrogen atmosphere. The Ultraviolet Photoelectron Spectroscopy (UPS) data were recorded on the Thermo Scientific NEXSA (ThermoFisher Nexsa). The gas discharge lamp was used for UPS, with helium gas admitted and the HeI (21.22 eV) emission line employed. UPS samples were prepared on a silicon substrate by spin coating. UV–vis absorption spectra were recorded using a SHIMADZU UV 3600I plus spectrophotometer. Photoluminescence Quantum Yield (PLQY) were gathered using an Edingburgh FL‐1000 fluorescence spectrometer. The morphology of the platform was observed using Scanning electron microscopy (SEM, Thermo Scientific Apreo 2C). Ionic strength was measured using static secondary ion mass spectrometry (TOF‐SIMS, ION TOF‐SIMS 5). Contact angles were recorded using a Dataphysics‐OCA20 contact angle‐measuring device. An infrared thermal imager (Fotric226s, Response band 8∼14 µm) was used to capture temperature imaging photos, and a contact‐type thermometer (UNI‐T, UT325) were used to record temperature changes. The photothermal experiment was performed using a xenon CME‐SL500 light source (AM 1.5G spectral filter). Calibration was performed using a Solar Power Meter (TES132) before each test to ensure accuracy. The sample (4 × 4 cm^2^) was placed horizontally 15 cm away from the light source, with a heat‐insulating pad positioned underneath. A thermocouple was attached to the fabric surface to measure the temperature, which was recorded via a computer. The electron spin resonance (ESR) spectra were measured by a Bruker A300 spectrometer.

### 4.3 Molecular Dynamics (MD) Simulation

Atomistic molecular dynamics simulations have been performed in the GROMACS [51] (version 2022.6) simulation package, using the General Amber force field (GAFF2). 80 chains of IEIC‐4F and IEICO‐4F molecules were randomly inserted into separate cubic boxes of around 10 nm. After thousands of steps of energy minimization, the systems were equilibrated under the NPT ensemble for 20 ns at relatively high temperature of 600 K to fully randomize the molecules and followed the production runs for another 20 ns. The final temperature was coupled to 298 K using the Nose‐Hoover method and the pressure was coupled to 1 atm using the Parrinello‐Rahman method. The cutoff scheme of 1.2 nm was implemented for the non‐bonded interactions, and the Particle Mesh Ewald method [52] with a Fourier spacing of 0.1 nm was applied for the long range electrostatic interactions. All covalent bonds with hydrogen atoms were constraint using the LINCS algorithm [53].

### 4.4 Testing of Fabric Properties

#### 4.4.1 Coating Preparation Method

Dissolve the organic semiconductor in dichloromethane at a concentration of 0.5 mg mL^−^^1^. Stir the solution thoroughly for 6 h until fully dissolved, then apply it onto the fabric surface via dip‐coating. Subsequently, allow the fabric to air‐dry naturally to obtain the organic semiconductor photothermal fabric, with a surface loading of about 0.25 g m^−2^ for the organic semiconductor on the fabric. Calculation of organic semiconductor loading: Before dip‐coating, the dry weight of the original fabric was accurately measured using a high‐precision balance. Then, a solution of organic semiconductor in dichloromethane was applied onto the fabric surface via dip‐coating. Afterward, the fabric was air‐dried in a fume hood for 12 h to ensure complete removal of dichloromethane (boiling point: 39.7°C, highly volatile). The dry weight of the fabric was measured again, and the loading amount was subsequently calculated. The air permeability test was conducted using the instrument SDL Atlas M021A USA, in accordance with the standard GB/T 5453. The sample pressure difference was 200 Pa, and the test head area was 20 cm^2^. The moisture permeability is measured according to GB/T 12704.1‐2009 standard. The test is conducted under conditions of relative humidity of 90%, temperature of 38°C, and air flow of 0.3–0.5 m/s. The water color fastness was tested using the commercial clean machine SW‐24E, Zhejiang San Gong Ren Instrument Co., Ltd. The perspiration color fastness was tested using the Perspiration Tester YG631W, Zhejiang San Gong Ren Instrument Co., Ltd. The rubbing color fastness was tested using the Rubbing Color Fastness Tester Y571D, Zhejiang San Gong Ren Instrument Co., Ltd., under conditions of 65.3% humidity and 21.1°C temperature. The tensile curve was tested using a universal testing machine (UTM5105). The color difference (Δ*E*) of the fabrics was tested using a colorimeter (KonicaMinolta CM‐5).

### 4.5 Antibacterial Test Method

Pick an appropriate amount of mature colonies and inoculate them into 5 mL of liquid medium. Culture at 37°C for 6–8 h until the OD600 reaches approximately 0.5. Adjust the pH to 7.4 using phosphate‐buffered saline (PBS) and dilute the bacterial suspension with PBS to a concentration of 10^6^ cfu/mL. Transfer 5 mL of the bacterial suspension into a 12 mL culture tube. Immerse the sample in the bacterial suspension and incubate in a constant‐temperature shaker at 37°C under xenon lamp illumination. After 24 h, collect the bacterial culture from the suspension and perform plate counting to determine the bactericidal rate.

### 4.6 Cytotoxicity Assay

Sterilize and extract the samples and obtain conditioned medium after 24 h. When the cells (L929/HaCaT) are in the logarithmic growth phase, seed the cell suspension (5000 cells/well) into a 96‐well plate. Then incubate the plate in a culture chamber (under conditions of 37°C and 5% CO_2_) for 24 h. After 24 h, add the conditioned medium and culture for 24, 48, and 72 h, respectively. After modeling, add 10 µL of CCK‐8 solution to each well (avoid generating bubbles in the wells, as they may affect OD value readings). Incubate the plate in the culture chamber for 2 h. Measure the absorbance at 450 nm using a microplate reader.

### 4.7 Ethical Approval Statement

Approved and confirmed by the Ethics Committee of the Health Science Center, Shenzhen University, an ethical review was conducted for the part of this study (Study number: M202600265) involving the photothermal effect of fabrics on skin. The volunteer, Mr. Zisuo Zhang, agreed to all skin tests related to the photothermal effect of the fabrics with informed consent.

## Conflicts of Interest

The authors declare no conflicts of interest.

## Supporting information




**Supporting File**: adma72886‐sup‐0001‐SuppMat.docx.

## Data Availability

The data that support the findings of this study are available in the supplementary material of this article.
