# Supplementary material for: Chromatic‐Invariant Photothermal Fabrics Enabled by Narrow‐Bandgap Organic Semiconductors for Wearable Solar Energy Harvesting
Source: Adv Mater. 2026 Mar 22;38(23):e19334. doi: 10.1002/adma.202519334 (PMC13103635; doi:10.1002/adma.202519334)
Supplement: Supplementary file 1 — Supporting File: adma72886‐sup‐0001‐SuppMat.docx. [file ADMA-38-e19334-s001.docx]

Supporting Information

**Chromatic-Invariant Photothermal Fabrics Enabled by Narrow-Bandgap Organic Semiconductors for Wearable Solar Energy Harvesting**

Jingshuai Zhu, Jianming Chen,* Shiyu Liao, Jiaxin Zheng, Shiguo Chen, Yuanfeng Wang,* Xungai Wang*

Dr. J. Zhu, Prof. S. Chen, Dr. Y. Wang

College of Materials Science and Engineering,

Shenzhen University,

Shenzhen, 518060, China

*E-mail: [wangyuanfeng@szu.edu.cn](mailto:wangyuanfeng@szu.edu.cn)

Dr. J. Zhu, Dr. J. Chen, S. Liao

Joint Research Centre for Fiber Innovations and Renewable Materials (JRC-FIRM),

School of Fashion and Textiles,

The Hong Kong Polytechnic University,

Kowloon, 999077, Hong Kong

*E-mail: [jianming.chen@polyu.edu.hk](mailto:jianming.chen@polyu.edu.hk)

Dr. J. Chen

Research Institute for Intelligent Wearable Systems (RI-IWEAR),

School of Fashion and Textiles,

The Hong Kong Polytechnic University,

Kowloon, 999077, Hong Kong

Dr. J. Zheng

School of Advanced Materials,

Peking University, Shenzhen Graduate School,

Shenzhen, 518055, China

Prof. X. Wang

Zhejiang Sci-Tech University, College of Textile Science and Engineering, Hangzhou, 310016, China.

*Email: xwang@zstu.edu.cn

**Experimental section**

**Synthesis**

IEIC-4F. Compound 1 (135 mg, 0.1 mmol), compound 2 (115 mg, 0.5 mmol), pyridine (0.6 ml), and chloroform (30 ml) were added to a three-necked round-bottom flask. The mixture was then deoxygenated with nitrogen for 20 min and stirred for 15 h. After cooling to room temperature, the mixture was poured into methanol (100 ml) and filtered. The residue was purified by silica gel column chromatography using petroleum ether: chloroform (1:1.5) as the eluent, yielding a black solid (130 mg, 73%). ^1^H NMR (500 MHz, CDCl_3_): *δ* 8.76 (s, 2H), 8.54 (m, 2H), 7.67 (m, 2H), 7.60 (s, 2H), 7.48 (m, 4H), 7.19 (m, 8H), 7.11 (m, 8H), 2.79 (m, 4H), 2.59 (m, 8H), 1.76 (m, 4H), 1.59 (m, 18H), 1.30 (m, 28H), 0.87 (m, 24H). ^13^C NMR (125 MHz, CDCl_3_): *δ* 185.71, 157.89, 157.14, 155.12, 153.94, 153.05, 152.93, 150.85, 149.25, 145.15, 141.60, 140.55, 139.78, 137.47, 137.10, 136.21, 135.15, 134.04, 133.46, 128.17, 127.42, 124.20, 120.76, 117.65, 114.65, 114.47, 113.88, 113.78, 112.25, 112.10, 69.20, 62.73, 38.88, 35.17, 33.36, 31.98, 31.31, 30.96, 29.30, 28.72, 28.14, 25.21, 22.62, 22.19, 13.71, 10.14. MS (MALDI-TOF): m/z 1776.1 (M^+^). Anal. Calcd for C_114_H_114_N_4_O_2_S_4_F_4_: C, 77.08; H, 6.47; N, 3.15. Found: C, 77.23; H, 6.51; N, 3.06.

IEICO-4F. Compound 1 (136 mg, 0.1 mmol), compound 2 (115 mg, 0.5 mmol), pyridine (0.6 ml), and chloroform (30 ml) were added to a three-necked round-bottom flask. The mixture was then deoxygenated with nitrogen for 20 min and stirred for 15 h. After cooling to room temperature, the mixture was poured into methanol (100 ml) and filtered. The residue was purified by silica gel column chromatography using petroleum ether: chloroform (1:1.5) as the eluent, yielding a black solid (126 mg, 71%). ^1^H NMR (500 MHz, CDCl_3_): *δ* 8.69 (s, 2H), 8.51 (m, 2H), 7.62 (m, 4H), 7.48 (m, 4H), 7.19 (m, 8H), 7.11 (m, 8H), 4.11 (m, 2H), 2.58 (m, 8H), 1.86 (m, 4H), 1.55 (m, 16H), 1.30 (m, 28H), 0.92 (m, 24H). ^13^C NMR (125 MHz, CDCl_3_): *δ* 185.54, 157.20, 156.30, 155.02, 154.39, 153.77, 152.04, 146.04, 140.96, 140.05, 137.17, 135.33, 135.02, 133.73, 130.50, 128.56, 127.54, 126.85, 122.64, 119.67, 117.05, 113.57, 111.18, 73.26, 67.00, 62.24, 38.57, 35.24, 30.70, 30.33, 29.48, 28.10, 25.98, 22.86, 21.97, 21.57, 13.08, 10.11. MS (MALDI-TOF): m/z 1779.6 (M^+^). Anal. Calcd for C_112_H_110_N_4_O_4_S_4_F_4_: C, 75.56; H, 6.23; N, 3.15. Found: C, 75.31; H, 6.33; N, 3.27.

**Calculation of the photothermal conversion efficiency**

Weigh out a specific mass of IEIC-4F and IEICO-4F. The *η* was assessed using the established classical method.^S1^ Based on the total energy balance for this system:

$\sum_{i} m_{i}c_{pi}\frac{dT}{dt}=Q_{s}-Q_{loss}$

*m_i_* (IEIC-4F: 9.2 mg, IEICO-4F: 11.8 mg) and *C_pi_* (IEIC-4F: 2.44 J (g ℃)^−1^, IEICO-4F: 1.99 J (g ℃)^−1^, Figures S7-8) are the mass and heat capacity of system components, respectively. *Q_s_* is the photothermal heat energy inputted by irradiating NIR laser to samples, and *Q_loss_* is thermal energy lost to the surroundings. Upon reaching its maximum temperature, the system attains a state of equilibrium:

$Q_{s}=Q_{loss}={hS\Delta T}_{max}$

where *h* is heat transfer coefficient, *S* is the surface area, *ΔT_max_* is the maximum temperature change. The *η* can be calculated using the following equation:

$\eta=\frac{{hS\Delta T}_{max}}{I(1-{10}^{-A_{808}})}$

where *I* is the laser power (1.0 W cm^-2^) and *A*_808_ is the absorbance of the samples at the wavelength of 808 nm (IEIC-4F: 0.49, IEICO-4F: 0.78, Figure S9). In order to obtain the *hS*, a dimensionless driving force temperature *θ* is introduced as follows:

$\theta=\frac{T-T_{surr}}{T_{max}-T_{surr}}$

where *T* is the temperature of samples, *T_max_* is the maximum system temperature (IEIC-4F: 193.5℃, IEICO-4F: 213.3℃, Figure S10), and *T_surr_* is the initial temperature (IEIC-4F: 25.5℃, IEICO-4F: 25.6℃, Figure S10). The sample system time constant *τ_s_* as follows:

$\tau s=\frac{\sum_{i} m_{i}c_{pi}}{hS}$

Thus $\frac{d\theta}{dt}=\frac{1}{\tau s}\frac{Qs}{{hS\Delta T}_{max}}-\frac{\theta}{\tau s}$. When the laser is turned off, *Q_s_* = 0, $\frac{d\theta}{dt}=-\frac{\theta}{\tau s}$, and $t=-\tau sln \theta$. Therefore, *hS* could be calculated from the slope of cooling time vs *ln θ*. τs is (IEIC-4F: 13.7 s, IEICO-4F: 12.8 s, Figures S11). The results show that IEIC-4F and IEICO-4F exhibited an impressive *η* of 40.7% and 41.3%, respectively.

Calculated the solar-weighted absorptance (SWA) using the AM 1.5G solar irradiance spectrum (ASTM G173-03) as the weight function.

SWA=$\frac{\int_{\lambda1}^{\lambda2} A\left( \lambda\right)\cdot E_{AM1.5}(\lambda)d\lambda}{\int_{\lambda1}^{\lambda2} E_{AM1.5}(\lambda)d\lambda}$

Where *A*(λ) is the absolute absorptance at wavelength λ, and *E*_AM1.5_(λ) is the solar irradiance λ.

**Figure S1.** UV-Vis (a) absorption spectra and (b) normalized absorption spectra of IEIC-4F and IEICO-4F in the solution (0.01 mg/ml, in chloroform).

**Figure S2.** UV-Vis (a) absorption spectra and (b) normalized absorption spectra of IEIC-4F and IEICO-4F in the film (prepared from 0.5 mg/ml chloroform solution).

**Figure S3.** UPS data of the IEIC-4F film.

**Figure S4.** UPS data of the IEICO-4F film.

**Figure S5.** TGA data of the IEIC-4F solid. IEIC-4F exhibits decomposition temperatures (*T*_d_, corresponding to 5% weight loss) of 329℃.

**Figure S6.** TGA data of the IEICO-4F solid. IEICO-4F exhibits decomposition temperatures (*T*_d_, corresponding to 5% weight loss) of 327℃.

**Figure S7.** Differential scanning calorimetry curve of IEIC-4F.

**Figure S8.** Differential scanning calorimetry curve of IEICO-4F.


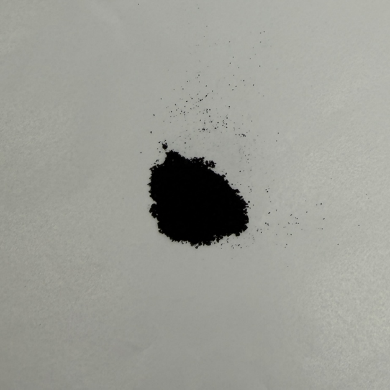


**Figure S9**. UV-Vis absorption spectra of IEIC-4F, IEICO-4F solids and image of the IEICO-4F solid.

**Figure S10**. Cooling curve of IEIC-4F and IEICO-4F solids.

**Figure S11.** Cooling time-ln*θ* curve.

**Figure S12.** UV-Vis absorption spectra of IEIC-4F and IEICO-4F on the cotton fabric.

**Figure S13.** The temperature of cotton fabric with varying IEICO-4F mass per unit area, recorded by a thermocouple.

**Figure S14.** Linear fit between irradiation intensity and fabric temperature (0.25 g m⁻^2^), the temperature values were recorded by a thermocouple.


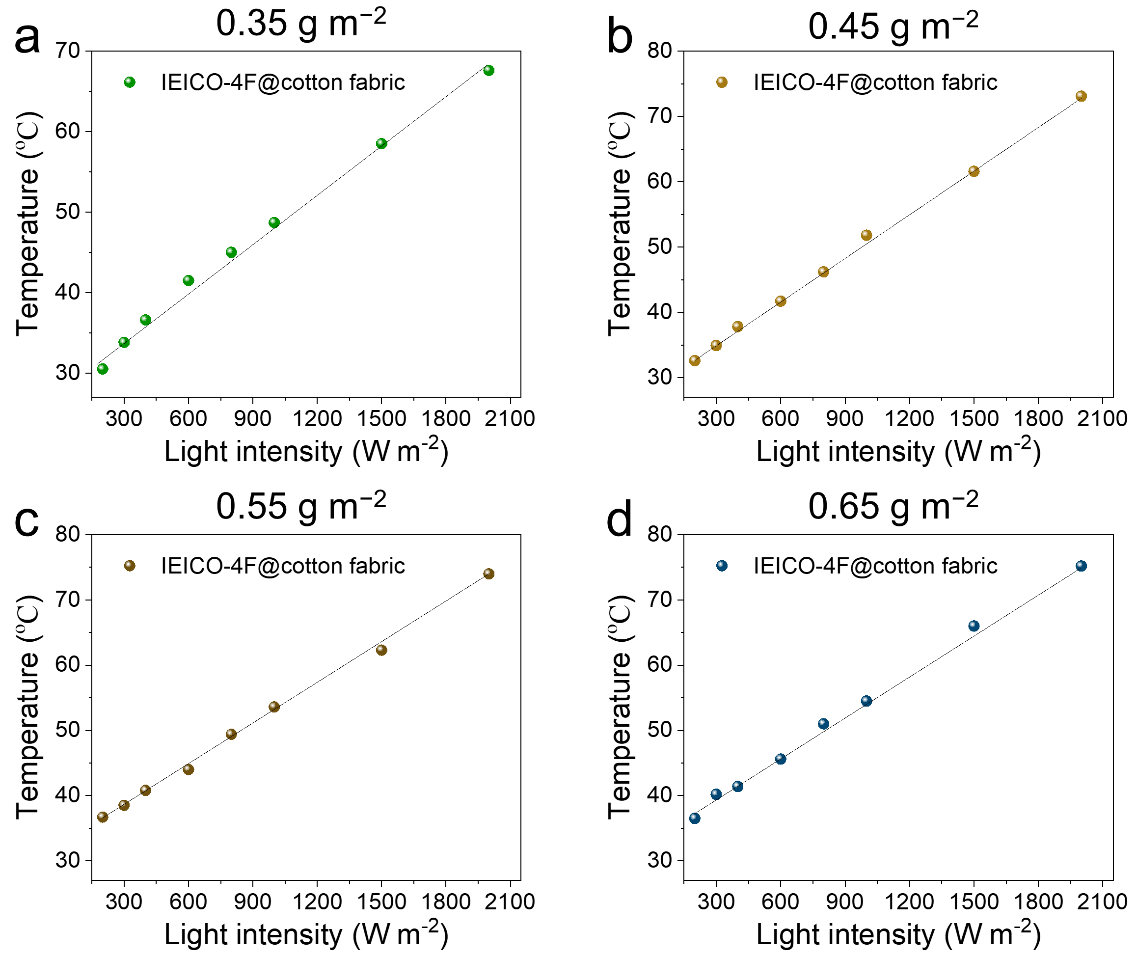


**Figure S15.** Linear fit between irradiation intensity and fabric temperature (0.35 g m⁻^2^ − 0.65 g m⁻^2^), the temperature values were recorded by a thermocouple.


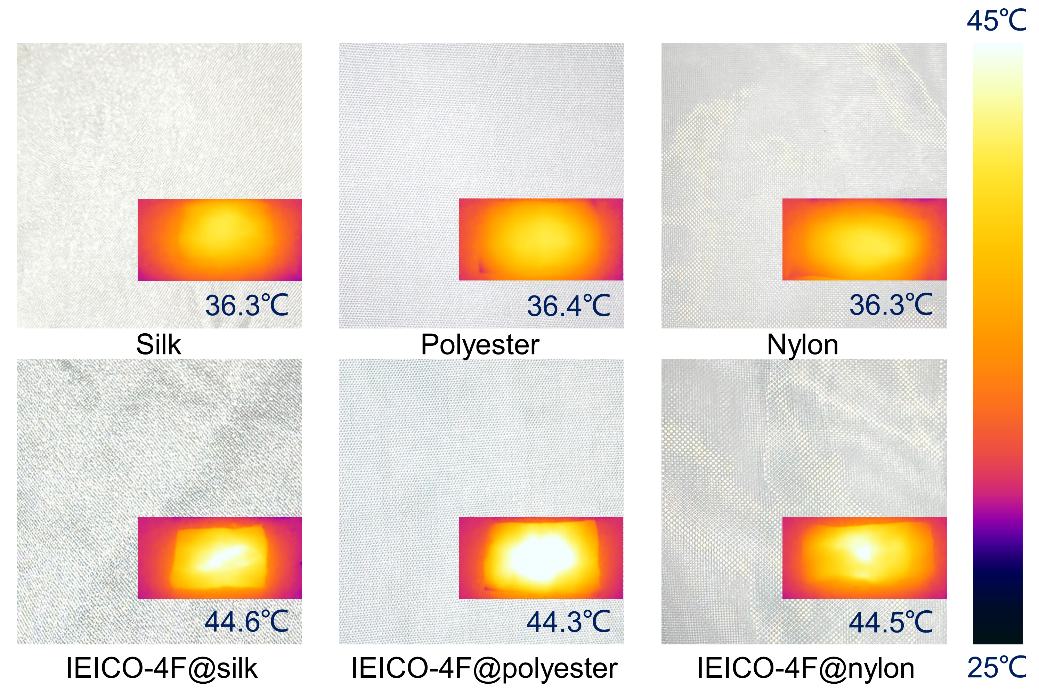


**Figure S16.** Different types of fabrics under one standard sunlight irradiation (mean of three experiments), the images were captured by a thermal imager, and the temperature values were recorded by a thermocouple.

**Figure S17.** TOF-SIMS elemental images of F^−^, CN^−^, S^−^, which was used as an indicator of IEICO-4F on the cotton fabric.


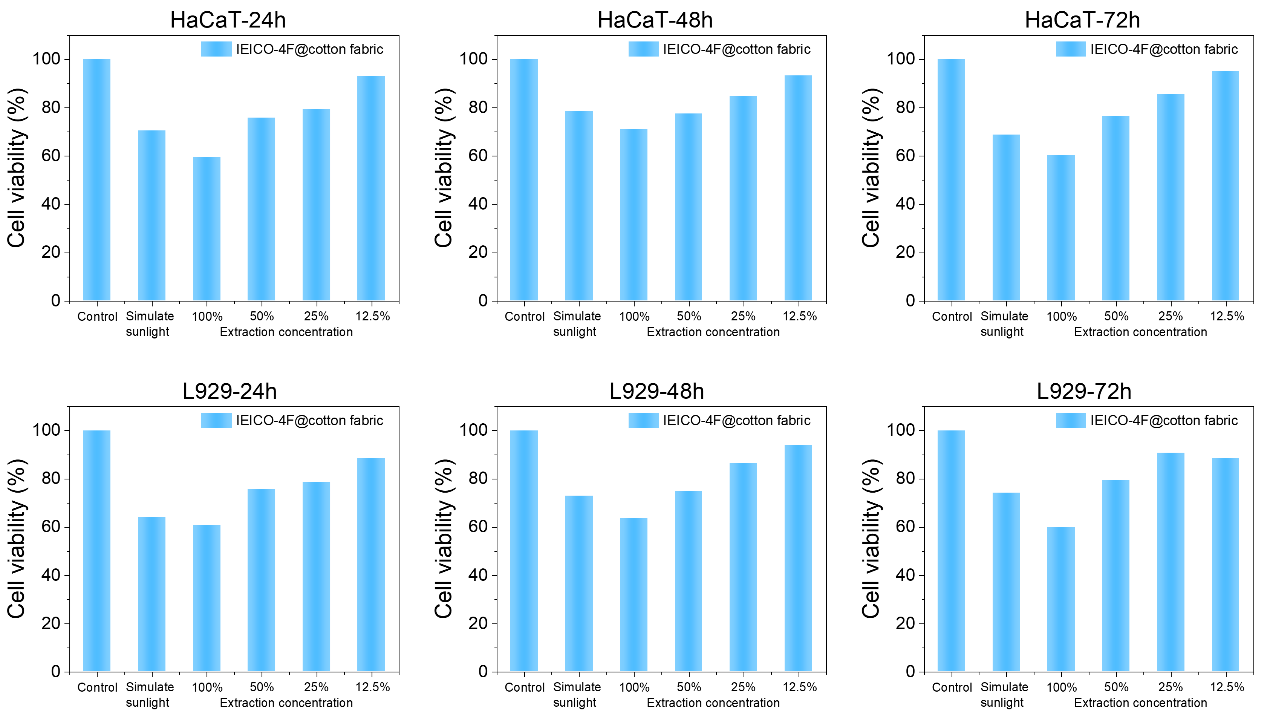


**Figure S18.** Cell viability of human keratinocytes (HaCaT) and mouse fibroblasts (L929) under different conditions (mean of three experiments).

**Figure S19.** Detailed *I*_solar_ and *T*_ambient_ in Beijing and Calgary.

**Figure S20.** Real-time temperature of the simulated skin covered by blank cotton fabric, IEIC-4F@cotton fabric, and IEICO-4F@cotton fabric over a duration of 5h under sunlight in Beijing and Calgary.

**Figure S21.** Linear fit between irradiation intensity and the temperature values (recorded by a thermocouple) of the three fabrics.

**Table S1.** Photothermal temperature of three fabrics measured by the thermocouple under different light intensities (after five minutes, averaged over three measurements).

|  | 0 W m^−2^ | 400 W m^−2^ | 600 W m^−2^ | 800 W m^−2^ | 1000 W m^−2^ |
| --- | --- | --- | --- | --- | --- |
| IEICO-4F@cotton fabric | 24.5°C | 33.3°C | 37.1°C | 41.0°C | 44.7°C |
| IEIC-4F@cotton fabric | 24.5°C | 32.5°C | 36.0°C | 39.5°C | 43.0°C |
| Blank cotton fabric | 24.6°C | 29.1°C | 31.0°C | 33.1°C | 35.2°C |

**Table S2.** Color parameters of blank cotton and IEICO-4F@cotton with different colors (Δ*E*=[(Δ*L*∗)^2^+(Δ*a*∗)^2^+(Δ*b*∗)^2^]^0.5^).

|  | *L** (D65) | *a** (D65) | *b** (D65) | △*E* |
| --- | --- | --- | --- | --- |
| Blank cotton (white) | 78.91 | 10.02 | −1.8 |  |
| IEICO-4F@cotton (white) | 76.68 | 8.27 | −2.08 | 2.85 |
| Blank cotton (red) | 40.6 | 50.37 | 25.64 |  |
| IEICO-4F@cotton (red) | 39.09 | 48.63 | 24.84 | 2.43 |
| Blank cotton (yellow) | 70.82 | 17.69 | 58.67 |  |
| IEICO-4F@cotton (yellow) | 70 | 16.59 | 57.49 | 1.81 |
| Blank cotton (blue) | 46.14 | −1.6 | −35.78 |  |
| IEICO-4F@cotton (blue) | 46.4 | −1.96 | −35.08 | 0.83 |
| Blank cotton (green) | 61.49 | −0.73 | 5.42 |  |
| IEICO-4F@cotton (green) | 60.61 | −1.29 | 5.01 | 1.12 |
| Blank cotton (black) | 22.21 | −0.26 | −0.79 |  |
| IEICO-4F@cotton (black) | 21.58 | −0.5 | −1.02 | 0.71 |

| Test Item | | Standard | Requirement | Evaluation Item | Result | Conclusion |
| --- | --- | --- | --- | --- | --- | --- |
| Washing Color Fastness  (Grade) | | GB/T 3921-2008  Method A (1) | - | Color Change | 4 | Compliant |
| Water Color Fastness (Grade) | | GB/T 5713-2013 | ≥3 | Color Change | 4 | Compliant |
|  |  |  | ≥3 | Staining - Acetate | 4-5 | Compliant |
|  |  |  |  | Staining - Cotton | 4-5 |  |
|  |  |  |  | Staining - Polyamide | 4-5 |  |
|  |  |  |  | Staining - Polyester | 4-5 |  |
|  |  |  |  | Staining - Acrylic | 4-5 |  |
|  |  |  |  | Staining - Wool | 4-5 |  |
| Perspiration Color Fastness (Grade) | Acid | GB/T 3922-2013 | ≥3 | Color Change | 4 | Compliant |
|  |  |  | ≥3 | Staining - Acetate | 4-5 | Compliant |
|  |  |  |  | Staining - Cotton | 4-5 |  |
|  |  |  |  | Staining - Polyamide | 4-5 |  |
|  |  |  |  | Staining - Polyester | 4-5 |  |
|  |  |  |  | Staining - Acrylic | 4-5 |  |
|  |  |  |  | Staining - Wool | 4-5 |  |
|  | Alkaline |  | ≥3 | Color Change | 4 | Compliant |
|  |  |  | ≥3 | Staining - Acetate | 4-5 | Compliant |
|  |  |  |  | Staining - Cotton | 4-5 |  |
|  |  |  |  | Staining - Polyamide | 4-5 |  |
|  |  |  |  | Staining - Polyester | 4-5 |  |
|  |  |  |  | Staining - Acrylic | 4-5 |  |
|  |  |  |  | Staining - Wool | 4-5 |  |
| Rubbing Color Fastness (Grade) | Dry Rubbing | GB/T 3920-2008 | ≥3 | Warp Direction | 4 | Compliant |

**Table S3.** Color Fastness of IEICO-4F@cotton fabric.

**Table S4.** Photothermal temperature of IEICO-4F@cotton fabric, IEICO-4F@silk, IEICO-4F@polyester, and IEICO-4F@nylon after repeated washing at 65°C, 30 min each time recorded by the thermocouple (after five minutes, averaged over three measurements).

|  | 10 times | 20 times | 30 times |
| --- | --- | --- | --- |
| IEICO-4F@cotton fabric | 44.5°C | 43.6°C | 41°C |
| IEICO-4F@silk | 44.7°C | 43.2°C | 40.5°C |
| IEICO-4F@polyester | 44.1°C | 42.7°C | 40.3°C |
| IEICO-4F@nylon | 44.2°C | 42.3°C | 39.6°C |

**Table S5.** Colony Forming Unit (CFU) counts and average values (1000 W m⁻^2^ simulated solar irradiation).

|  | Plate | Dilution factor | CFU Prorata | Average value | Antibacterial rate |
| --- | --- | --- | --- | --- | --- |
| *Escherichia coli* | Control | 10000 | 49 | 510,000 | 0 |
|  |  |  | 57 |  |  |
|  |  |  | 47 |  |  |
| *Escherichia coli* | Blank cotton fabric | 10000 | 49 | 503,333.333 | 1.3% |
|  |  |  | 56 |  |  |
|  |  |  | 46 |  |  |
| *Escherichia coli* | IEICO-4F@cotton fabric | 10000 | 7 | 93,333.333 | 81.7% |
|  |  |  | 10 |  |  |
|  |  |  | 11 |  |  |
| *Staphylococcus aureus* | Control | 10000 | 37 | 396666.667 | 0 |
|  |  |  | 40 |  |  |
|  |  |  | 42 |  |  |
| *Staphylococcus aureus* | Blank cotton fabric | 10000 | 33 | 386666.667 | 2.5% |
|  |  |  | 41 |  |  |
|  |  |  | 42 |  |  |
| *Staphylococcus aureus* | IEICO-4F@cotton fabric | 1000 | 16 | 18333.333 | 95.3% |
|  |  |  | 17 |  |  |
|  |  |  | 22 |  |  |

**Table S6.** Colony Forming Unit (CFU) counts and average values (IEICO-4F@cotton fabric).

|  | Conditions | Dilution factor | CFU Prorata | Average value | Antibacterial rate |
| --- | --- | --- | --- | --- | --- |
| *Escherichia coli* | Control | 10000 | 109 | 1123333.333 | 0 |
|  |  |  | 112 |  |  |
|  |  |  | 116 |  |  |
| *Escherichia coli* | ROS scavenger, 37°C incubation | 10000 | 94 | 1013333.333 | 9.8% |
|  |  |  | 100 |  |  |
|  |  |  | 110 |  |  |
| *Escherichia coli* | 1000 W m⁻^2^ irradiation, ROS scavenger, 37°C incubation | 10000 | 22 | 250000.000 | 77.7% |
|  |  |  | 25 |  |  |
|  |  |  | 28 |  |  |
| *Escherichia coli* | 500 W m⁻^2^ irradiation, 37°C incubation | 10000 | 19 | 226666.667 | 79.8% |
|  |  |  | 23 |  |  |
|  |  |  | 26 |  |  |
| *Escherichia coli* | 300 W m⁻^2^ irradiation, 37°C incubation | 10000 | 27 | 293333.333 | 73.9% |
|  |  |  | 29 |  |  |
|  |  |  | 32 |  |  |
| *Escherichia coli* | Fabric after 1000 W m⁻^2^, 20 h irradiation | 10000 | 23 | 233333.333 | 79.2% |
|  |  |  | 23 |  |  |
|  |  |  | 24 |  |  |
| *Staphylococcus aureus* | Control | 10000 | 90 | 1006666.667 | 0 |
|  |  |  | 99 |  |  |
|  |  |  | 113 |  |  |
| *Staphylococcus aureus* | ROS scavenger, 37°C incubation | 10000 | 85 | 876666.667 | 12.9% |
|  |  |  | 88 |  |  |
|  |  |  | 90 |  |  |
| *Staphylococcus aureus* | 1000 W m⁻^2^ irradiation, ROS scavenger, 37°C incubation | 1000 | 69 | 73000.000 | 92.7% |
|  |  |  | 73 |  |  |
|  |  |  | 77 |  |  |
| *Staphylococcus aureus* | 500 W m⁻^2^ irradiation, 37°C incubation | 1000 | 43 | 51000.000 | 94.9% |
|  |  |  | 53 |  |  |
|  |  |  | 57 |  |  |
| *Staphylococcus aureus* | 300 W m⁻^2^ irradiation, 37°C incubation | 1000 | 85 | 92666.667 | 90.8% |
|  |  |  | 92 |  |  |
|  |  |  | 101 |  |  |
| *Staphylococcus aureus* | Fabric after 1000 W m⁻^2^, 20 h irradiation | 1000 | 69 | 75000.000 | 92.5% |
|  |  |  | 75 |  |  |
|  |  |  | 81 |  |  |

**Table S7.** Comparison of the *η* of some typical photothermal materials.

| Materials | *η* | Test condition | References |
| --- | --- | --- | --- |
| IEICO-4F | 41.3% | 808 nm laser | This work |
| Ag-MOF-1* | 51.8% | 808 nm laser | S2 |
| Ag-MOF-2* | 36.2 % |  |  |
| Bi_2_Se_3_/Cu_2_-xS | 54.6% | 808-nm laser | S3 |
| TTF@1a | 35.65% | 808-nm laser | S4 |
| TTF@1b | 40.65% |  |  |
| MFPP (MoS_2_) | 46.86% | 808 nm NIR laser | S5 |
| Carbon spheres | 54.2% | 808 nm laser | S6 |

**References**

S1. Lu. B, Chen. Y, Li. P, Wang. B, Mullen. K, Yin. M. *Nat. Commun.* **2019**, *10*, 767.

S2. P.-Y. Liao, J.-X. Li, J.-C. Liu, Q. Xiong, Z.-Y. Ruan, T. Li, W. Deng, S.-D. Jiang, J.-H. Jia, M.-L. Tong *Angew. Chem. Int. Ed.* **2024**. *63*, e202401448.

S3. Y. Liu, S. Pan, W. Xia, P. Qin, W. Wang, Q. Liu, X. Chen, L. Ma, S. Ding, Q. Wang, *Sci. Adv.* **2025**, *11*, eadt2884.

S4. X.-W. Li, Y. Huang, H. Fang, H.-Y. Wang, H. Sun, M.-H. Yu, Y. Du, Z. Chang, X.-H. Bu, *Nano Lett.* **2025**, *25*, 1093.

S5. A. Sharma, D. Bhalothia, R. Vankayala, A. Bagaria, *Sci. Rep.* **2025**, *15*, 26028.

S6. C. Wei, X. Jin, C. Wu, A. Brozovic, W. Zhang, *Diam. Relat. Mater.* **2022**, *126*, 109048.
